# Lycopene Scavenges Cellular ROS, Modulates Autophagy and Improves Survival through 7SK snRNA Interaction in Smooth Muscle Cells

**DOI:** 10.3390/cells11223617

**Published:** 2022-11-15

**Authors:** Ayed A. Shati, Refaat A. Eid, Mohamed Samir A. Zaki, Youssef A. Alqahtani, Saleh M. Al-Qahtani, Harish C. Chandramoorthy

**Affiliations:** 1 Department of Child Health, College of Medicine, King Khalid University, Abha 62529, Saudi Arabia; 2 Department of Pathology, College of Medicine, King Khalid University, Abha 62529, Saudi Arabia; 3 Department of Anatomy, College of Medicine, King Khalid University, Abha 62529, Saudi Arabia; 4 Department of Histology and Cell Biology, College of Medicine, Zagazig University, Zagazig 31527, Egypt; 5 Stem Cell and Regenerative Medicine Research Unit, College of Medicine, King Khalid University, Abha 62529, Saudi Arabia; 6 Department of Microbiology & Clinical Parasitology, College of Medicine, King Khalid University, Abha 62529, Saudi Arabia; 7 Institute for Healthcare Education and Translational Sciences (IHETS), W. Marredpally, Telengana 500026, India

**Keywords:** lycopene, small ns-RNA, smooth muscle cells, reactive oxygen species, anti-oxidant enzymes, proinflammatory cytokines

## Abstract

The chance of survival rate and autophagy of smooth muscle cells under calcium stress were drastically improved with a prolonged inclusion of Lycopene in the media. The results showed an improved viability from 41% to 69% and a reduction in overall autophagic bodies from 7% to 3%, which was well in agreement with the LC3II and III mRNA levels. However, the proliferation was slow compared to the controls. The fall in the major inflammatory marker TNF-α and improved antioxidant enzyme GPx were regarded as significant restoration markers of cell survival. The reactive oxygen species (ROS) were reduced from 8 fold to 3 fold post addition of lycopene for 24 h. Further, the docking studies revealed binding of lycopene molecules with 7SK snRNA at 7.6 kcal/mol docking energy with 300 ns stability under physiological conditions. Together, these results suggest that Lycopene administration during ischemic heart disease might improve the functions of the smooth muscle cells and 7SK snRNA might be involved in the binding of lycopene and its antioxidant protective effects.

## 1. Introduction

Prolonged stress-mediated oxidant injury results in irreversible tissue damage [1]. Especially, specialized cells, such as smooth muscle cells (SMCs), are vulnerable to stress and loss of function due to hypoxia, ROS, protein aggregates and DNA damage [2]. A wide range of clinical conditions, including chronic progressive inflammatory diseases (Atherosclerosis), pulmonary fibrosis is attributed to the loss of SMCs function due to cellular stress [3]. Though SMCs are varied and derived from percussor cells originating from different anatomical locations, their response to stress and resultant pathological features also diverge [2]. Further, the mechanism of stress-mediated cellular dysfunction along with involvement of small non-coding RNA has not been sufficiently addressed [4]. The cellular features due to stress may be almost the same as those resulting in gross failure of SMCs [5]. Autophagy associated with SMCs are well acknowledged as one of the manifestations associated with loss of function and thereby cell death [5]. Though autophagy is an evolutionary mechanism to maintain cellular homeostasis, defective autophagy becomes irreparable and induces apoptosis resulting in the loss of the cells or cellular functions [6]. Therefore, evidence of autophagy indeed seems to be necessarily an important indication associated with SMCs stress and largely mediated by metabolic intermediates, which are further proposed to be epigenetically regulated [7].

Though cellular stress is varied, the pathways in which cells succumb to death or are non-functional largely depend on the induction of proinflammatory cytokine mediated by the infiltration of macrophages [8] and diminished secretion of cellular antioxidant enzymes [9]. There are well characterized multifunctional transcriptional regulator small non-coding RNAs (small nc-RNAs) controlling nuclear activity required for various local and basal genes involved in cellular development etc. [10]. There are many other mediators of destructive cellular functions including necroptosis [11] mitochondrial ROS-mediated apoptosis [12]. However, many of these stress pathways usually end up in nonfunctional autophagy or apoptosis leading to failure of SMCs. Mechanistic pathways that could lead to ROS-mediated induction of cell dysfunction are well characterized in proliferative diseases. While small or long nc-RNA’s might modulate these pathways leading to autophagy or mitophagy. There are reports pertaining to the epigenetic and transcriptional factors including recent studies on micro-RNA on the mitochondrial damage leading to mitochondrial Reactive Oxygen Species (mROS) generation and thereby death [13]. These factors can be cytokines or other chemical messengers, such as Ca or Mg, leading to high mROS cascade of events leading to cell death [14]. Generally, it is assumed that TNF-α is a proinflammatory cytokine known to induce irreversible cellular damage in response to cellular stress signals [1]. The presence of proinflammatory cytokine in-turn promotes calcium [Ca^2+^] mediated cellular stress resulting in the elevated production of cytosolic ROS (cROS) [12]. Release of stored [Ca^2+^] from endothelium also results in overloading of mitochondria, a known [Ca^2+^]_c_ buffer resulting in the permanent loss of mitochondrial membrane potential promoting apoptosis [15]. The role of antioxidant enzymes and oxidant chelators are well proven concepts with protective effect over cell homeostasis [16]. The relationship of immediate reduction in the production of antioxidant enzymes in response to the increased proinflammatory cytokines are recently documented [17] and becomes a point of intervention in the management of the chronic inflammatory diseases, such as Atherosclerosis or reperfusion injury [18]. There are few known antioxidants that are currently used in the drug format [19]. Many naturally occurring compounds are known for their antioxidant potential, and Vitamin D is one such well-studied natural compound [20]. Lycopene is another naturally occurring material that is known for extended bio-activity [21,22,23], including antioxidant property [24]. Some studies suggest that Lycopene can protect oxidation of DNA, proteins and lipids [25,26]. Lycopene has been used as a nutritional supplement alongside multivitamins [16]. It may be noted that many studies show natural compounds to scavenge the oxidants through antioxidant factors, while these antioxidants are controlled or transcriptionally regulated by small non-coding RNAs. Hence, in the current investigation, Lycopene was supplemented in the media that is used in conditioning the SMCs post hypoxic stress. The same was documenting in-silico binding of the lycopene with small non-coding RNAs involved in cellular functions.

## 2. Materials and Methods

The study was undertaken at the Stem Cell and Regenerative Medicine Research Unit and Electron Microscopy Center at the Department of Pathology, College of Medicine, King Khalid University, Abha, Saudi Arabia. Ethical clearance was obtained from the King Khalid University ethical committee, College of Medicine, approval letter REC #2015-03-07 for the collection of umbilical cord/cord blood. Human umbilical vein smooth muscle cells (HUVSMC) were retrieved from stem cells and the regenerative medicine research unit LN_2_ storage and propagated with DMEM/F12 media supplemented with 5% fetal bovine serum, 2 mM of glutamine, and 1× penicillin and streptomycin. Cells were maintained at 37 °C at 5% CO_2_ and 95% humidity in a CO_2_ incubator.

### 2.1. Design of the Study

The early passage HUVSMCs were subjected to stress by oxygen glucose deprivation method described elsewhere [27]. The stressed cells were then restored with regular media and oxygen conditions with or without lycopene in the media for the next 48 h with replenishing media after 24 h. Briefly the HUVSMCs were seeded in 24-well tissue culture treated plates at a density of 1 × 10^5^ cells/well. After overnight growth with confluence of 60 to 70%, the cells were washed once with 0.1% phosphate buffered saline (without ca and mg) and the media was replenished with glucose-free basal media, which was pre-bubbled with 100% N_2_ for a minimum of 30 min. The plates were sealed with sealing tape (multi-well plate sealing tape) to prevent exchange of gases and incubated for 5 h at 37 °C. The restoration of the normal growth conditions was by replacing the glucose free media with complete growth medium with or without Lycopene supplementation and conditioning for overnight at 37 °C at 5% CO_2_ and 95% humidity. Based on the Lycopene results, the docking studies were performed for Lycopene and small non-coding RNAs that are physiologically known to induce transcriptional regulation in scavenging the oxidants.

### 2.2. Viability and Cell Proliferation

Viability of the HUVSMCs were measured by the trypan blue method [28]. To the 10 μL of washed cells each from hypoxia, control, recovery, recovery + Lycopene which was resuspended in 100 μL of Phosphate buffered saline (PBS) was mixed 90 μL of 0.4% trypan blue solution and then vortexed. A total of 10 μL of the mixture was loaded in a hemocytometer, and the cells were counted from the four corner squares at 40× magnification under a light microscope (Binocular Optical Microscope, Olympus ^®^ Waltham, MA 02453, United States. Cells that excluded the trypan blue (viable cells) were counted against total cells to calculate the viable cells per ml using the formula.

$$Viable\;Cells\;\left(\%\right)=\frac{Total\;number\;of\;viable\;cells\;per\;\mathrm{mL}}{Total\;number\;of\;cells\;per\;\mathrm{mL}}\times 100$$

The HUVSMCs proliferation was measured by 3-(4,5-dimethylthiazol-2-yl)- 2,5-diphenyltetrazolium bromide (MTT) assay described elsewhere [29] with modifications to suit the current experimental model. Briefly, the cells cultured with or without hypoxia media and recovery or recovery + Lycopene were checked for proliferation for 24,48,72 and 96 h. To the wells 15 μL of 5 mg/mL MTT was added and incubated for 3.5 h followed by addition of 150 μL of dimethyl sulfoxide and absorbance was read at 560 nm with reference at 640 nm.

### 2.3. PCR for Autophagic Markers, TNF-α and GPx

Autophagy was enumerated by extraction of total RNA from the cells by Qiagen Total RNA extraction. RT-PCR will be performed using the primer sets for LC3A, LC3B and β-Actin given below Table 1. The RT-PCR cycling conditions were 15 min at 95 °C followed by 40 cycles of 15 s at 95 °C, 15 s at 58 °C and 20 s at 72 °C. Melting was done at 60–95 °C with an increment of 0.5 °C and holding time of 5 s per step [30].

### 2.4. Cytosolic ROS Determination

The ROS was determined by Reactive Oxygen Assay Kit (Cat # CA1410) Solarbio Life Sciences, Beijing, China according to the manufacturer instructions. Briefly, the cells that were either stressed or recovered were treated with DCFH-DA (10 μM) for 20 min at 37 °C post removal of the media. The cells were then washed with serum free media and read at dual wavelengths of 488 and 525 nm using a fluorescence plate reader. The amount of ROS was calculated compared to the cell control and positive control given in the kit for the quality control process.

### 2.5. Transmission Electron Microscopy (TEM)

All specimens were immediately immersed in 2.5% paraformaldehyde -glutaraldehyde in 0.1M sodium cacodylate buffer, pH 7.4, and placed in a thermal box cooled to 4 °C for 2 h. The samples were further centrifuged at 5000× *g* and pellets were washed two times with sodium cacodylate buffer. They were post-fixed in 1% osmium tetroxide in a sodium cacodylate buffer and then dehydrated in an ascending series of ethanol and embedded in Super’s resin. Ultrathin sections stained with uranyl acetate and lead citrate were examined by transmission electron microscope (JEM-1011, Jeol Co., Tokyo, Japan) at 80 KV in the EM-Unit, College of Medicine, King Khalid University [32].

### 2.6. Non-Coding RNA Interaction with Lycopene

Structure retrieval: Structure of non-coding RNA molecule was retrieved from the PDB database. NMR structure of stem-loop 4 from the human 7SK snRNA in complex with arginine (2KX8) was retrieved and processed before docking analysis. Lycopene molecule was retrieved from the PubChem database (446925). Structure was converted to 3D mol2 format using BIOVIA Discovery Studio Visualizer.

RNA-ligand Docking: Docking calculations were performed using the SiBDOCK module from SiBioLead server (www.sibiolead.com, accessed on 12 September 2022) which uses Autodock-Vina docking algorithm for docking calculation. Docking grid box set to the maximum 120 to ensure the whole RNA is covered for blind docking.

Molecular Dynamics Simulations: Molecular Dynamics (MD) simulations of RNA-lycopene complex was performed using MD simulation module developed at SiBioLead LLP which uses GROMACS simulation algorithm for MD simulations. Briefly, RNA-lycopene complex was first typed with OPLS forcefield. The RNA-lycopene complex was immersed in a triclinic simulation box containing Simple Point Charge (SPC) water molecules and NaCl as counterions. For maintaining physiological conditions, a further 150 mM NaCl, and 20 mM MgCl was added to the simulation system. The Lycopene molecule was preprocessed using the AmberTools and Acpype package. Simulation system was energy minimized for 5000 steps using the Steepest Descent method. Before the actual MD run, the whole system was equilibrated for 300 ps using NVT/NPT protocol. MD simulation was conducted for 300 ns in duplicates after which simulation trajectories were analyzed using GROMACS in-built results analysis package. Results were visualized using BIOVIA Discovery studio.

### 2.7. Statistics

All the experiments were repeated at least three individual times. The data are summation from the three repeats unless specified. The data are expressed as mean ± SE, and statistical significance was evaluated via one-way analysis of variance (ANOVA). A *p*-value of <0.05 was considered statistically significant depending on the post hoc test, such as Kruskal–Wallis and Bonferroni’s Multiple Comparison, unless other tests specified. Data were plotted with Graph Pad Prism v.6.0. (San Diego, CA, USA).

## 3. Results

**Figure 1:**
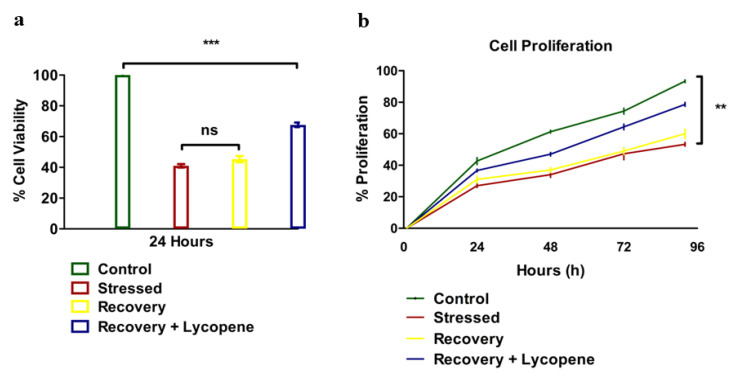
(**a**) Cell viability of HUVSMCs (n = 3, (*p* < 0.0145)). (**b**) Cell Proliferation of HUVSMCs during stress, recovery and recovery + Lycopene (n = 3, (*p* < 0.0001)). All the tests were repeated at-least three times at different time points for consistency. (** *p* < 0.01, *** *p* < 0.001).

The results of the viability of SMC’s were greatly affected by the stress and even after the reversal of stress by replacing the stressors with normal media, did not show immediate restoration of the dying cells. However, the cells which were supplemented with lycopene in the recovery media showed elevated viability compared to the stressed or recovery group *p* < 0.0145 (Figure 1a). The proliferation was also compromised (Figure 1b) with the stressors while recovery + lycopene supplementation clearly restored the maximum number of cells as observed by comparing the proliferation trend from 24 to 96 h (*p* < 0.0001).

**Figure 2:**
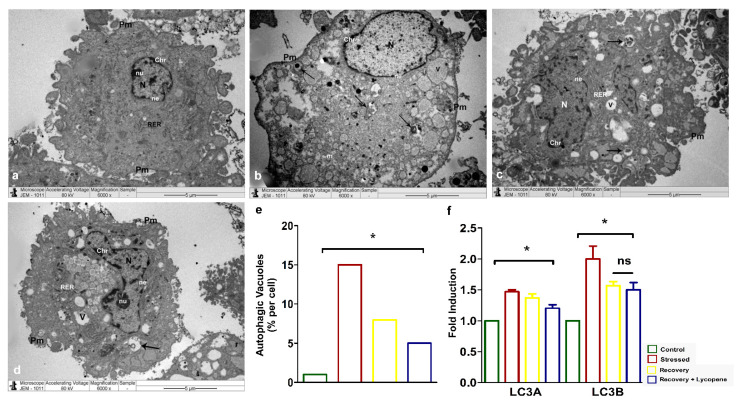
(**a**–**d**) TEM showing accumulation of autophagic vacuoles. (**e**) Quantitation of autophagic vacuoles in % (*p* < 0.0158). (**f**) LC3A and LC3B autophagic markers by RT-PCR (LC3a—*p* < 0.0187, LC3b—*p* < 0.0242). Abbreviations: N—Nucleus, nu—Nucleolus, ne—nuclear envelope, Pm—Plasma membrane, Chr—Chromatin materials, m—Mitochondria, RER—Rough endoplasmic reticulum, Arrows—Autophagic vacuoles. (* *p* < 0.05).

Secondly, the diminished cell viability and slow recovery of the SMCs to normal phenotype was assessed for autophagy by transmission electron microscopy (TEM). The cells which were under stress (Figure 2b,e) showed enhanced number of autophagic vacuoles (~15%) compared to the control (Figure 2a) while the recovery by lycopene + recovery media (Figure 2d,e) was significantly lower (~5%) than the recovery (Figure 2c,e) by plain media which showed (~8%) (*p* < 0.0158). The induction of autophagy and subsequent recovery by lycopene was confirmed by mRNA expression of the autophagic markers LC3A (*p* < 0.0187) and B (*p* < 0.0242) (Figure 2f) which very well correlated with the TEM autophagy (Figure 2a–e).

**Figure 3:**
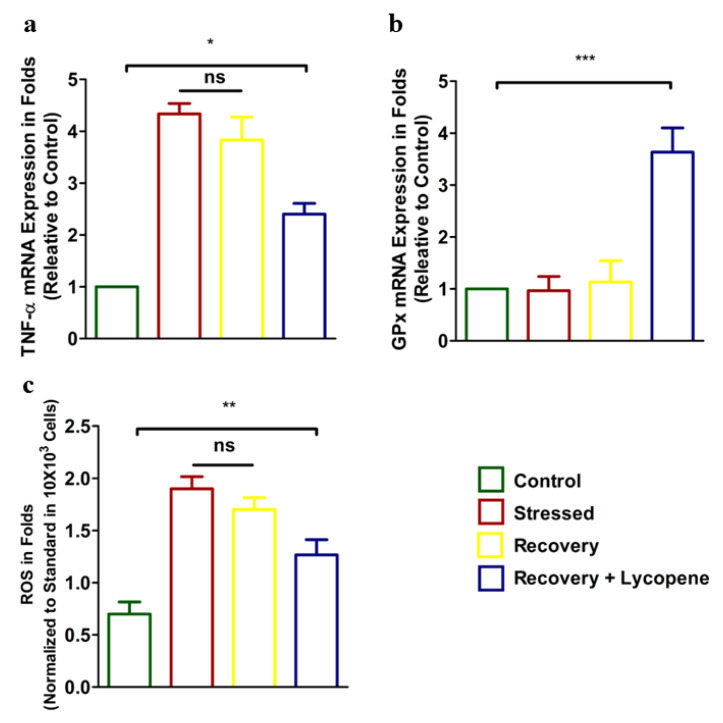
(**a**) mRNA expression of TNF-α. (*p* < 0.0429) (**b**) mRNA expression of GPx (*p* < 0.0072). (**c**) Cytosolic ROS levels (*p* < 0.0009) in control, stressed, recovery and recovery + Lycopene treated cells. (* *p* < 0.05, ** *p* < 0.01, *** *p* < 0.001).

Next, the expression of proinflammatory cytokine TNF-α correspondingly antioxidant enzyme GPx and ROS were enumerated. Results showed increased expression of TNF-α in the stressed group (Figure 3a). Addition of Lycopene to the media showed diminished TNF-α expression (*p* < 0.0429) compared to the stressed group. The results of anti-oxidant enzyme GPx which was low in the stressed group was dramatically recovered by 2.5 folds (*p* < 0.0072) compared to the stressed group (Figure 3b). Further, the ROS levels, which were correspondingly high in the stressed group, were chelated in the recovery + Lycopene group (*p* < 0.0009) and was within the observation of GPx that well validated our results (Figure 3c).

**Figure 4:**
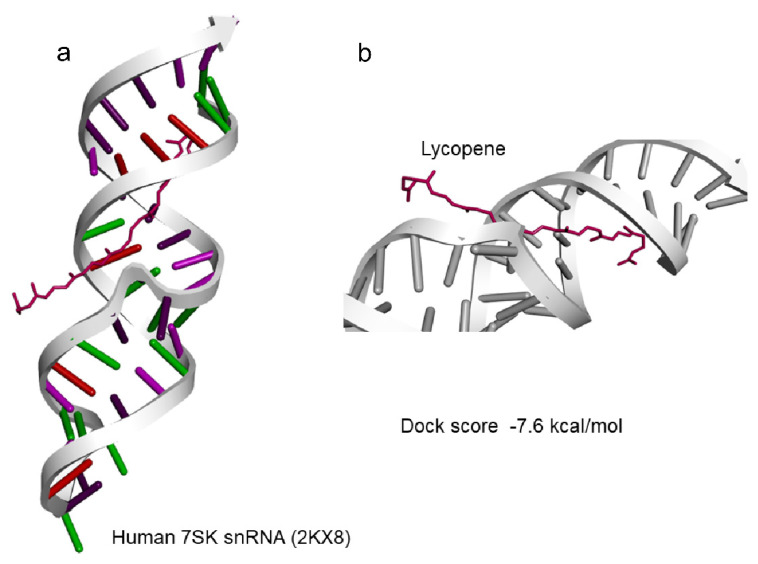
Docking of Lycopene to (**a**) 7SK snRNA (2KX8) and (**b**) with dock score of −7.6 kcal/mol.

**Figure 5:**
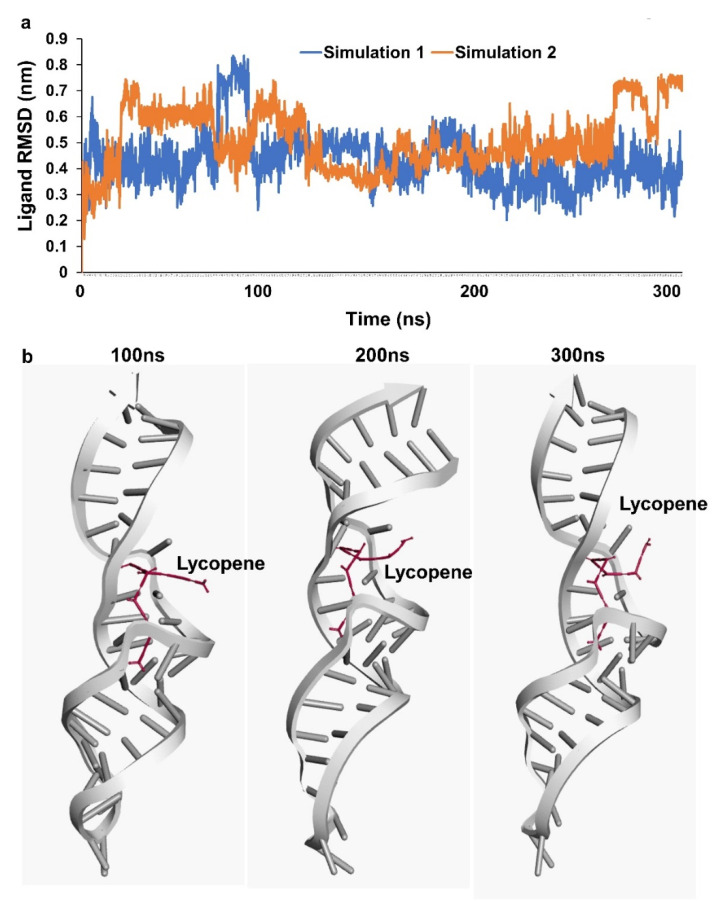
Simulation assessment (**a**) simulation trajectory analysis of RNA-lycopene complex. (**b**) stability of RNA-lycopene complex during simulation at 100, 200 and 300 ns.

Based on the results of lycopene effectively scavenging the oxidants, docking analysis for lycopene molecules and binding avidly to various target non-coding RNA was carried out. 7SK snRNA a well characterized small non-coding RNA was observed to be well docked with lycopene at a predicted docking energy of −7.6 kcal/mol (Figure 4a,b). The stability of the binding and the dynamics of the 7SK snRNA in the presence of lycopene molecule at 300 ns simulation in a solvated condition including water/ions and mimicking other physiological conditions as in nucleus did not show any disassociation (Figure 5a). Further analyzing the simulation results indicate, lycopene molecule binds stably and avidly to the non-coding RNA molecule (Supplementary Materials Video S1). Visualizing the simulation trajectories at different time points indicate that lycopene molecule binds stably with 7SK snRNA at the predicted position throughout 300 ns simulation (Figure 5b). The binding affinity of lycopene to 7SK snRNA, from the simulation trajectories show a consistent binding free energy for both the simulations, indicating an avid binding of lycopene to RNA (Supplementary Materials Figure S1).

## 4. Discussion

The overall results of addition of lycopene showed functional recovery of stressed SMCs toward survival was well in agreement with other studies on the same line which used other natural products, such as Vitamin D as supplement in the functional restoration of the SMCs [20]. The cell viability post stress and restoration of the viability versus proliferation was inversely related in the current observation. It may be assumed that the loss of the dead cells is compensated by the proliferation of the restored cells which could be evidenced by cellular turnover in the recovery and recovery + lycopene group. Some studies have shown irreversible autophagy due to prolonged stress while restoration along with mediators of anti-inflammatory and antioxidants have largely shown improved tissue regeneration and restoration of functional SMCs [33]. Current research findings also share this trend by showing improvement in antioxidant enzymes and reduction of proinflammatory cytokines, which grossly resulted in the low turnout of autophagy markers at mRNA levels confirmed by few autophagy bodies by TEM observation. Though, it is not known how exactly lycopene or other natural compounds, such as Vitamin D, induce anti-oxidants or reduce proinflammatory cytokines [16] it is well in agreement with other literature evidence which has used different cell types [34]. Further, we did dock studies to deduce whether lycopene interacts with short non-coding RNAs which could be an important factor regulating cell functions by gene activity or transcriptional regulation. To our surprise, 7SKsnRNA, a well characterized short RNAs [35] strongly bound with lycopene at physiological conditions, throws light on the reversible autophagy observed in SMCs. Our observation was in great agreement with growing evidence on the short nc-RNAs regulating functions of cells through transcriptional regulation of survival genes [36].

Autophagy is a well proved coordinated response to various cellular stressors, including hypoxia, growth factors or nutrient deprivation, cytosolic and mitochondrial ROS, damage of DNA, formation of protein aggregates due to damaged organelles or intracellular pathogens [37,38]. Many of these stressors are known to induce sequential events of cellular stress, which results in poor recovery of functional cell types as observed in current results. Therefore, supplements that mediate antioxidants or anti-inflammatory cytokines along with restoration of the proper nutrient supply is essential for restoration of the cells to normalcy. These observations were well correlated with studies that had been done similarly elsewhere with different cell models [39,40,41]. The results of the dock studies of lycopene with 7SKsnRNA revealed a possible regulation by stopping the translocation of 7SKsnRNP [42], thereby regulating the distress signals to switch for survival.

Since, it is well known that proinflammatory cytokines will induce ROS [43] as well as reduce the antioxidant enzymes making the cellular functions more complicated [44,45], it is well justified to use multiple factors for resolving stress-mediated cell damage [46]. Prolonged stress will be sufficient to kill many cells, however recovery with an antioxidant or anti-inflammatory inducer, such as 7SKsnRNA or other transcriptional regulators, are necessary for at least restoring the cells that are exposed to cellular stressors [47], as observed in the current study. It may be observed that lycopene is effective in reducing proinflammatory cytokine TNF-α and restoring GPx and scavenging the ROS. There are studies which delineate induction of ROS by cytokines or low ROS turnover in immune cells by cytokines [45], and it is not well known whether the cellular stress coordinates with other mechanisms in reducing the expression of antioxidants and therefore induces ROS or proinflammatory cytokines [48]. Physiological restoration of cells to normal phenotype by native cellular machinery [49] with optimal growth conditions may vary depending on the acute or chronic nature of the stress, while effective mediators, such as lycopene, will enhance cell recovery and function through induction snRNA [50]. Generally, the restoration of SMCs has been challenging compared to other cell types, owing to its anatomical location and function [51]. Current in-vitro results suggest that lycopene can restore the SMCs function while other studies have reported that lycopene can be absorbed by passive diffusion or by scavenger receptor class B type 1 (SR-B1), hence the investigation can be escalated to animal models [52]. Lycopene interacting and binding with small ncRNA opens new avenues in understanding elimination of oxidants and ROS-mediated stress observed in SMCs [53].

## 5. Conclusions

From the current observations it is clear that hypoxia induces oxidant (ROS) mediated SMC dysfunction. Results show reduction in GPx turnover and induction of proinflammatory cytokine TNF-α and thereby ROS skewing the cells to autophagy. Though the sequential order by which ROS is generated is not clear, addition of lycopene along with reversal of hypoxia conditions enhances cell recovery which was evidenced by sustained cell viability, enhanced proliferation and reduced autophagic bodies in the recovered cells. Further dock results of lycopene with well characterized 7SKsnRNA evidenced the possibility of regulation of cell survival. Therefore, addition of naturally occurring antioxidants, such as lycopene, can initiate reversal of stressed SMCs through scavenging of oxidants and inducing small nc-RNA to transcriptionally regulate cell survival.

## Figures and Tables

**Table 1:** RT-PCR Primer Sequences.

Target Gene	Primer Sequence F- Forward, R-Reverse	Amplicon Size (bp)
LC3A	F: 5ʹ-CGTCCTGGACAAGACCAAGT-3′ R: 5ʹ-CTCGTCTTTCTCCTGCTCGT-3′	181
LC3B	F: 5ʹ-AGCAGCATCCAACCAAAATC-3′ R: 5ʹ-CTGTGTCCGTTCACCAACAG-3′	187
β-Actin	F: 5ʹCAACTGGGACGACATGGAGAAAAT-3′ R: 5ʹ-CCAGAGGCGTACAGGGATAGCAC-3′	207

TNF-α (Hs00174128_mL), GPx (Hs00829989_gH) and GAPDH (Hs03929097_gl) were enumerated by Taq man Gene expression assay from applied biosystem (Foster City, CA, USA). The target mRNA was enumerated using 2−ΔΔCt method by normalizing to GAPDH expression. All the samples were tested in triplicates at least three times repeated at different time points unless specified [31].

## Data Availability

The data presented in this study are available upon reasonable re-quest from the corresponding author.

## Supplementary Materials

The following are available online at https://www.mdpi.com/article/10.3390/cells11223617/s1, Figure S1: MM-PBSA based binding free estimate analysis for lycopene to RNA, Video S1: 300 ns simulation video.
