# Supplementary material for: Lycopene Scavenges Cellular ROS, Modulates Autophagy and Improves Survival through 7SK snRNA Interaction in Smooth Muscle Cells
Source: Cells. 2022 Nov 15;11(22):3617. doi: 10.3390/cells11223617 (PMC9688495; doi:10.3390/cells11223617)
Supplement: Supplementary file 1 [file cells-11-03617-s001.zip › cells-1984045-supplementary.pptx]

## Slide 1
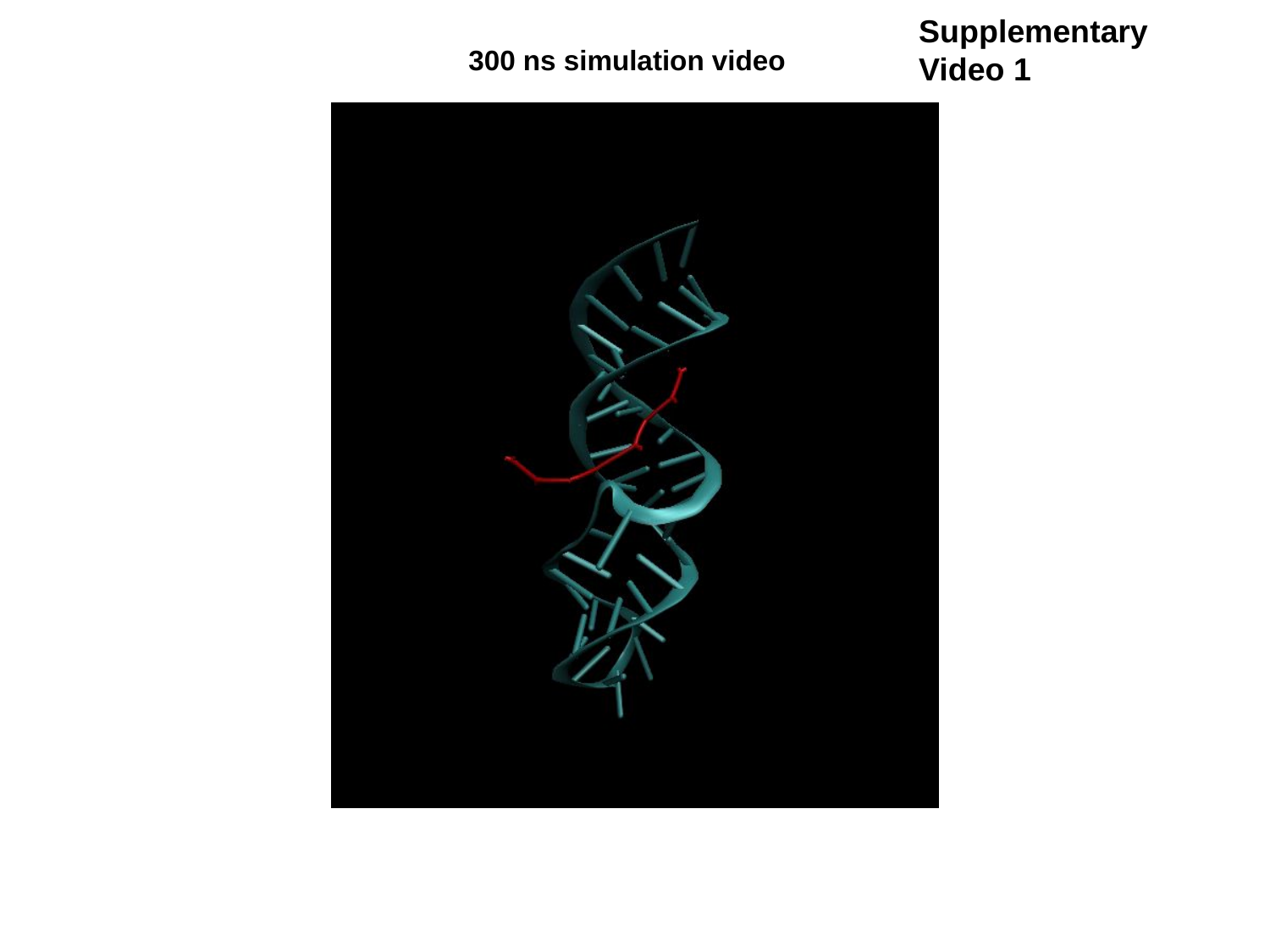

Supplementary Video 1
300 ns simulation video

## Slide 2
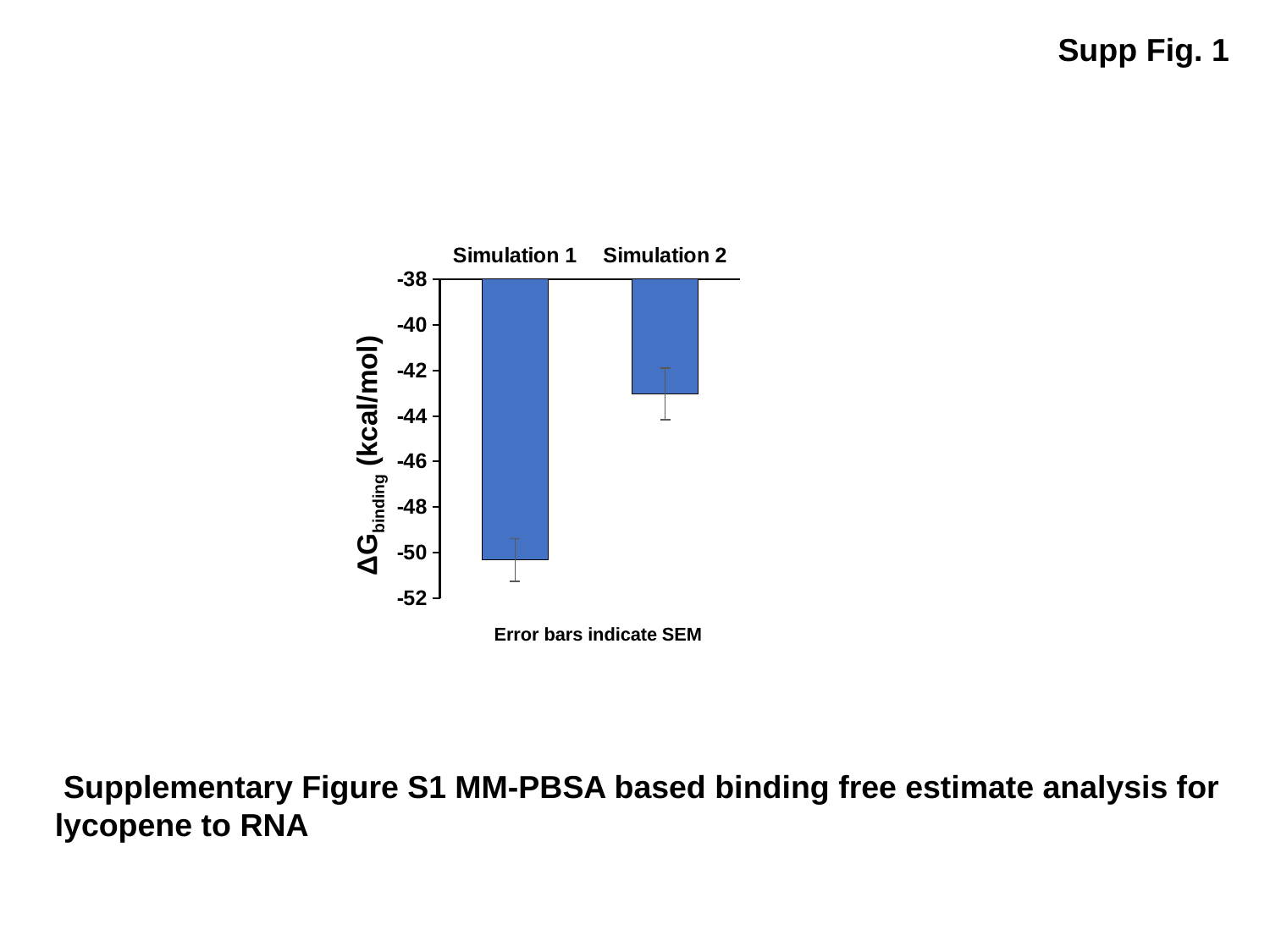

Supp Fig. 1
### Chart
| Category | |
|---|---|
| Simulation 1 | -50.32 |
| Simulation 2 | -43.04 |ΔGbinding (kcal/mol)
Error bars indicate SEM
 Supplementary Figure S1 MM-PBSA based binding free estimate analysis for lycopene to RNA
